# Patient-Centric Design of Topical Dermatological Medicines

**DOI:** 10.3390/ph16040617

**Published:** 2023-04-19

**Authors:** Rita Oliveira, Isabel F. Almeida

**Affiliations:** 1 FP-BHS—Biomedical and Health Sciences Research Unit, FFP-I3ID—Instituto de Investigação, Inovação e Desenvolvimento, Faculdade de Ciências da Saúde, Universidade Fernando Pessoa, Rua Carlos da Maia 296, 4200-150 Porto, Portugal; ritao@ufp.edu.pt; 2 UCIBIO—Applied Molecular Biosciences Unit, MedTech, Laboratory of Pharmaceutical Technology, Department of Drug Sciences, Faculty of Pharmacy, University of Porto, Rua Jorge Viterbo de Ferreira 228, 4050-313 Porto, Portugal; 3 Associate Laboratory i4HB—Institute for Health and Bioeconomy, Faculty of Pharmacy, University of Porto, Rua Jorge Viterbo de Ferreira 228, 4050-313 Porto, Portugal

**Keywords:** patient-centric, drug-product design, topical medicines, adherence, vehicles

## Abstract

Topical treatments are essential approaches to skin diseases but are associated with poor adherence. Topical vehicles have the primary purpose of ensuring drug effectiveness (by modulating drug stability and delivery, as well as skin properties) but have a marked impact on treatment outcomes as they influence patient satisfaction and, consequently, adherence to topical treatments. There is also a wide variety of vehicles available for topical formulations, which can complicate the decisions of clinicians regarding the most appropriate treatments for specific skin disorders. One of the possible strategies to improve topical-treatment adherence is the implementation of patient-centric drug-product design. In this process, the patient’s needs (e.g., those related to motor impairment), the needs associated with the disease (according to the skin lesions’ characteristics), and the patient’s preferences are taken into consideration and translated into a target product profile (TPP). Herein, an overview of topical vehicles and their properties is presented, along with a discussion of the patient-centric design of topical dermatological medicines and the proposal of TPPs for some of the most common skin diseases.

## 1. Introduction

Non-adherence to treatment is universally recognized as a public health problem. Non-adherence leads to suboptimal health outcomes, lower quality of life, and higher healthcare costs [1]. Poor adherence has been reported for several dermatological conditions [2,3,4,5]. The World Health Organization (WHO) recommends that the determinants of non-adherence are classified into five main dimensions: socioeconomic factors, health-care and system-related factors, therapy-related factors, condition-related factors, and patient-related factors. Topical treatments are widely used in dermatology and are the most commonly used therapeutic approaches [6]. However, several reports suggested low satisfaction with topical treatments. For instance, patients with psoriasis consider topical therapy to be one of the most negative aspects of the disease. Their satisfaction is significantly lower with this treatment compared to systemic treatments [7]. The rates of adherence to topical treatments are relatively low (50–70%) and have been related to poor cosmetic acceptability [8,9,10]. Satisfaction with topical treatment seems to be a key determinant of adherence [11], and this is the rationale for prioritizing these formulations for inclusion in patient-centric drug-product-design processes, thus contributing to the maximization of adherence. Iversen et al. suggested that the improvement of the vehicles through which topical treatments are applied has the potential to result in significant clinical and patient benefits [12]. Despite technological advances, commercial drug products and clinical prescriptions of compounding formulations are focused on a reduced number of vehicles.

This review aims to address a variety of topical vehicles, the process of patient-centric topical dermatological medicines’ design, and its relevance in dermatological treatments.

## 2. Vehicles/Bases Used in Topical Dermatological Treatments

Vehicles are mixtures of excipients that carry the drug to the administration site. Although the term vehicle is commonly used for any dosage form, according to the European Pharmacopoeia, it refers only to liquid formulations, while for semisolid dosage forms, the official designation is base [13].

Topical vehicles/bases, i.e., products intended for application on the skin, scalp, or nails, include solutions, emulsions, suspensions, ointments, creams, pastes, gels, foams, sticks, and powders. They are designed to be inert and cosmetically acceptable, and most have emollient and moisturizing properties. Creams and ointments are the commonly most used bases in the treatment of skin disorders [10,14].

Liquid vehicles include solutions, suspensions, and emulsions, with variable viscosity (Table 1) [15,16], and when they are applied topically, they are also known as lotions. Solutions are defined as liquid formulations, in which a solute (or solutes) is (or are) dispersed in a solvent at the molecular level. Solutions can be applied in several anatomic areas, such as the body skin, scalp, or nails. Shampoos are liquid preparations that are composed of a surfactant dispersion, suitable for scalp application.

Suspensions are liquid formulations in which insoluble solid particles are dispersed. Usually, the particles tend to settle, and agitation before use is required.

Emulsions are homogeneous two-phase liquid systems, obtained from the dispersion of immiscible liquids, wherein the internal phase is dispersed in droplets in the outer phase. Depending on the composition of the internal and external phases, they are classified as W/O emulsions (with oil as the outer and continuous phase) or O/W emulsions (with water as the outer and continuous phase). The water- and oil-phase components and the emulsifying system determine the type of emulsion and its occlusive properties. When they are semisolid, they are called creams.

Semisolid bases allow drug retention at the application site and are usually easily spread on the skin; most have lubricating and emollient properties. They consist of different types of bases that vary according to consistency and hydrophilicity/lipophilicity, namely, hydrophobic, absorption, emulsions, hydrophilic, hydrogels, pastes, and foams (Table 2) [15,16].

Ointments are one-phase preparations that comprise hydrophobic, absorption (or water-emulsifying), and anhydrous hydrophilic bases, with the common property of low water miscibility and an occlusive effect that varies with their composition. They have high viscosity and low spreadability, and are difficult to wash off. Furthermore, W/O creams are made of a lipophilic outer phase that incorporates water with the presence of W/O-type emulsifying agents. They are emollient and slightly occlusive, and their greasiness depends on the amount of oil phase (20–50%), which allows good absorption of liposoluble drugs, such as corticosteroids, retinoids, and hormones. Additionally, O/W creams are composed of an external water phase and O/W-type emulsifying agents. They are non-occlusive and non-oily, easily applied, and removable (from the skin and hair). Based on the type of surfactant, they can be divided into anionic and nonionic emulsions. Anionic emulsions are reproducible and stable but can be also irritants due to their components (e.g., sodium lauryl sulfate), and they may present some incompatibilities with the drugs incorporated. Four classic emulsions, presented in ascending order of fat content, are Lanette lotion, Beeler base cream, Lanette base cream, and hydrophilic ointment. Nonionic emulsions are suitable for sensitive skin, since they are composed of non-irritating emulgents with low fat contents and, therefore, exert milder effects on the skin.

Emerging emulsified vehicles/bases tend to be more compatible with the skin and less aggressive than the more frequently prescribed anionic emulsions/creams. Thus, the aims of current emulsions are to reduce of the number of ingredients, ensure the high quality and purity of ingredients, avoid irritating or photosensitizing substances, perfumes, and colorants, reduce the amount of preservatives, and ensure compatibility with the physiological pH of the skin. Glycoside emulsions/creams have a low fat content and include non-ionic and non-ethoxylated emulsifying agents that are compatible with the skin, such as sugar-based emulsifying agents (esters of glucose or sucrose; polyglyceryl stearates), which are better-tolerated [17]. They present very good organoleptic properties and they are moisturizing, fluid, and suitable for facial areas and sensitive or reactive skin [18]. Cream gels or emulgels also have very good skin tolerance, as they are composed mainly of water, a low-fat phase, and well-tolerated polymers (such as polyacrylate polymers) [19]. Water-in-silicon emulsions/creams have an outer phase composed of silicones instead of fats. They form a water-repellent film with no oily residue, present good cosmetic properties, and constitute non-comedogenic oil-free emulsions [20,21].

Gels are usually composed of a matrix of colloidal organic polymers that entrap the solvent (if they are water-based, they are called hydrogels) and drug. Inorganic polymers can also originate hydrogels with a semisolid consistency. Oleogels can be obtained through the jellification of liquid oils with a bivalent soap or another organogelator. Recently, several new organogelators were studied [22,23,24].

Pastes contain large amounts of insoluble powders in hydrophobic bases (the most common) or hydrophilic bases. Both present drying and absorbent effects.

Foams are liquids or semisolids in special pressurized packages with a propellant hydrocarbon, delivering the product through an actuated valve. They are easy to use on all skin surfaces without spreading and, in general, leave no residue on the skin. Continuous innovations have taken place in foam technology, which has moved from hydroethanolic-based formulations to aqueous or emulsion-based foams [25,26].

Solid vehicles are probably the least commonly used vehicles in topical applications. Powders are dry and fine solids and are frequently used for their drying and astringent effects [15,16]. Solid sticks are prepared by molding and can have different compositions, such as hydrophobic (a combination of waxes and oils), high-molecular-weight PEGs, or soaps (sodium stearate). All solid sticks have the advantage of high drug stability and sliding application of the drug.

Several authors have classified topical vehicles/bases according to their ingredients and properties [27,28,29,30,31] but, in general, they do not relate them to skin disorders or patient preferences. A patient-centric approach is crucial for obtaining maximum therapeutic effectiveness and is further discussed in the context of dermatological medicines.

## 3. Patient-Centric Topical-Medicine Design

**Figure 1:**
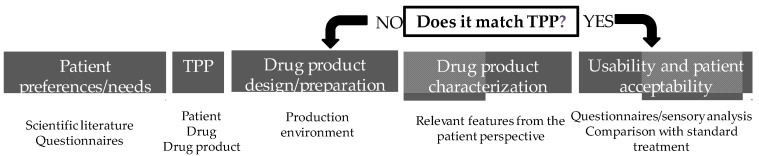
Patient-centric drug-product-design stepwise approach.

Regulatory authorities are increasingly placing patients at the center of pharmaceutical development. The European Medicines Agency (EMA, Amsterdam, Netherlands) has issued guideline/reflection papers for pediatric [32,33] and older populations [34], while the United States Food and Drug Administration (FDA, Silver Spring, MD, USA) has developed a series of guidance documents on patient-focused drug development, with the primary goal of incorporating the patient’s voice in drug development and evaluation [35], as well as other research [36,37,38]. The International Council for Harmonization of Technical Requirements for Pharmaceuticals for Human Use (ICH, Geneva, Switzerland) also published a guideline to advance patient-focused drug development [39]. Patient-centric drug-product design (PCDPD, Wanchai, Hong Kong) can be defined as the process of identifying the comprehensive needs of individuals or target patient populations and utilizing the identified needs to design pharmaceutical drug products that provide the best overall benefit-to-risk profile for specific target patient populations for the intended duration of treatment [40]. Patient-centric drug-product design is a stepwise approach (Figure 1) that starts with the evaluation of patient preferences and needs to obtain the necessary patient input to define the target product profile (TPP) [41,42]. It has been applied to the design of oral pediatric formulations [43], solid dosage forms [44,45], and medications for the elderly, as well as topical formulations, such as an emulgel for psoriasis [46]. Although PCDPD can be applied at any stage of the drug-development lifecycle, this paper focuses on the definition of TPP for topical formulations. Since topical medicines are often associated with poor satisfaction, they are an obvious choice for the application of the PCDPD process. Target product profiles are defined according to insights into patients, drugs, and drug products collected with questionnaires or based on scientific research. The drug product is then designed, prepared, and characterized concerning relevant features from the patient’s perspective. At this point, the matching of its features to the TPP is evaluated and reformulation takes place, if necessary. After obtaining the optimized formulations, the translation into a higher level of patient satisfaction with the topical treatment in comparison with standard treatment should be verified in a sample population (Figure 1). The putative advantages include the satisfaction of unmet needs and higher satisfaction with treatment, which in turn encourages better medication adherence and therapeutic outcomes [41].

In a PCDPD approach, the patient’s perspective can be included in product development at various stages, such as defining outcomes in clinical evaluation by establishing the most significant symptoms, the tolerability of adverse effects, risk–benefit assessment, or preferences for improving acceptability and adherence [39]. Even in the early development phases, communication between multiple stakeholders during the product development chain would help to meet patients’ needs, improving their quality of life [47].

When describing a drug product and its attributes for pharmaceutical development, several dimensions must be taken into account to obtain a medicine that complies with quality, efficacy, and safety requirements. The definition and prioritization of critical attributes are performed through risk management, establishing the greatest impact on the final product. The acceptability and usability assessment of the product is the main key to patient-centric drug-design approaches. Stegemann et al. developed a roadmap to achieve the TPP, which can be further integrated into the Quality-by-Design process during the development phase, re-formulation, or other life-cycle phases of the drug product [48]. Providing a TPP suitable for some groups of skin disorders, adverse effects, adherence, usability, and acceptability represent the patient’s preferences and needs, which may also affect the course of the disease.

### 3.1. Patient Preferences and Needs Regarding Topical Medicines

#### 3.1.1. Patient- and Disease-Related Needs in Dermatology

The selection of vehicles/bases for dermatological treatments should consider the type of skin lesion. Very dry lesions are lichenified (with thickening, darkening, pleating of the skin) and xerotic. They require very occlusive vehicles to soften the skin. Dry lesions (with scaling) represent a large proportion of skin pathologies and also require occlusive vehicles, reducing the fat content, especially in the face and capillary zones. Subacute lesions present intermediate characteristics between those of dry and wet lesions: scaling, excoriations, and crusts. Vehicles/bases should be emollient but not overly occlusive to reduce macerated skin. Wet lesions present erythema and edema, but are not exudative. In these cases, vehicles/bases should not be occlusive, in order to avoid edema (with low or no fat content). Furthermore, W/O emulsions are recommended for wet and interdigital lesions and pastes present drying effect. For exudative lesions, drying vehicles/bases are used (often containing antiseptics) and when they stop exuding, they are treated as wet lesions. Solutions or hydrogels are the vehicles of choice [31].

The anatomic site determines the skin properties, namely the thickness, and vehicles need to be customized [30]. The palmoplantar region presents thicker skin, while facial and pleated areas are more permeable and, therefore, need different fat contents. Glabrous areas, such as the trunk and limbs (when little hair-bearing) are considered intermediate in terms of thickness. For nail pathologies, nail varnishes allow the easy application and high retention of drugs. For hairy sites, the vehicles should facilitate the application and removal of the product with reduced consistency and lipophilicity. The most common vehicles are hydroalcoholic solutions, shampoos, hydrogels, and light and volatile oils. In hyperkeratotic lesions with crust formation (as in psoriasis or seborrheic dermatitis) due to the dry effects of solutions and when emulsions are not easily applied to the scalp, heavy-oily solutions are applied overnight. In skin conditions, such as ichthyosis, psoriasis, and atopic dermatitis, in which the epidermal barrier is damaged, excessive friction should be avoided during the application of topical treatments. Topical preparations in these cases should present specific attributes, such as ease of spread, and high pressure should not be applied during their application [49].

Table 3 summarizes the recommended vehicles/bases according to anatomic site.

Furthermore, it is important to take into account the skin type during vehicle/base selection for facial application. Facial skin and sensitive areas are critical, especially when facing prolonged topical treatment. Dry skin needs some occlusion to increase hydration, while oily skin needs the opposite. For sensitive or reactive skin, it is important to select non-irritant excipients, such as non-ionic emulsifiers, and a more inert composition, such as cream gels or glycoside emulsions. Table 4 suggests some vehicles that can be chosen, especially for facial skin.

Ultimately, the purpose of a topical vehicle/base is to carry and deliver a drug, contribute to its stability, retain the substance at the site of action, and facilitate its skin permeation. However, regarding skin disorders, the vehicle can play a role that is complementary to that of the drug [50] and contribute to the therapy by modulating the skin’s water content [51,52,53], improving the lipid–skin barrier [54,55], or regenerating skin cells [56]. Van Zuuren et al. conducted a systematic review of five randomized clinical trials to assess the effects of moisturizers on eczema and found that the moisturizing effect produced better results when added to the drug than a placebo vehicle or no moisturizer [57]. The use of enriched topical vehicles with non-drug substances to improve the skin barrier can improve dermatitis and decrease the use of corticosteroids [58]. Hydrophilic bases and cleansing lotions showed a better tolerance to benzoyl peroxide formulations by reducing skin irritation [59,60]. The positive effect of the vehicle/base on skin-disorder treatment still needs to be further explored.

In addition to their skin condition, the needs of the patient must also be accounted for when prescribing the treatment regimen. When patients present with impairments in motoric function (e.g., rheumatoid arthritis), the ability to open closure systems, squeeze tubes, rub formulations onto the skin, or reach less accessible areas can be impaired. In these cases, fluid vehicles are preferred, and the package should be easy to handle. The elderly is also a special population presenting a variable degree of frailty. In cases of blindness, packages should present braille inscriptions, whereas if a mild degree of cognitive impairment is present, the instructions for use should be easy to understand, and the packaging should be simple [61].

#### 3.1.2. Patient Preferences for Topical Medicines

Several studies underlined that patient preferences need to be considered when prescribing topical treatments to maximize adherence and improve clinical outcomes [12,62,63,64,65,66,67]. The topical application procedure includes four steps: (a) removal from a container (pick-up), (b) the primary sensation upon the first contact with the skin, (c) the secondary sensation during spreading on the skin, and (d) the final impression, through skin residue. Each patient applies semisolid/liquid formulations to the skin with a slightly different motion and their mechanical and sensory features are closely looked at by patients during topical application [68]. The mechanical properties of different topical anti-psoriatic medicines have been shown to vary substantially, demonstrating that topical vehicles can be perceived in very different ways during their application on the skin [14]. Vehicles also differ in their hydrophilic/lipophilic character, as mentioned before, which results in differences between the sensations they create in the skin. Vehicle excipients can also influence skin moisturization and tolerability [31]. All these differences can influence patient satisfaction and justify, at least partially, their preferences.

A limited number of studies have addressed patient preferences regarding topical products. Patient preferences can vary according to the skin disease and the location of the affected area [69]. For example, the preferences of acne patients (*n* = 19) were found to be markedly different from those of patients suffering from atopic dermatitis (*n* = 18) [69]. Regarding lesion location, patients might avoid using ointments in locations where their clothes might come into contact with the medicated area to avoid staining their clothes. A preference for more fluid forms for hairy regions, such as the scalp, is also common. Furthermore, in one study, age group, ethnicity, and gender were also shown to influence preferences for particular vehicles/bases (*n* = 404) [70]. The patients younger than 40 years preferred lotions, while patients aged over 40 preferred creams. An analysis based on gender showed that females preferred creams, while males preferred lotions and ointments. A strong preference for ointments was found in black-skinned patients, while for white-skinned patients, cream was the preferred form. Few vehicles/bases were included in the survey, and the reasons behind these preferences were not studied. Since it was established that skin condition and anatomic location influence vehicle preference, the results obtained without controlling these variables have limited value.

Fisher et al. studied the influence of ethnicity on vehicle preference for the scalp and found that compared with Caucasian patients (*n* = 100), African American patients (*n* = 100) mostly prefer ointments for treating scalp conditions over other topical preparations. A general assumption is that ointment will prevent hair shaft frizzing and drying [71].

Concerning vehicle-type preferences, acne patients were shown to tend to prefer washes, creams, and lotions [62,72]. A conjoint analysis conducted to determine patient preferences for topical antibiotic treatments for acne found that the patients preferred gel formulations to lotions (*n* = 67) [73]. Interestingly, this analysis revealed that the patients’ experiences using the medications had a substantial effect on their reported preferences. While hydrogels were not popular choices before treatment, they became the preferred dosage form by far after ending the treatment. A new tretinoin lotion formulated with a polymeric emulsion technology for the uniform delivery of micronized tretinoin and moisturizing excipients was associated with fewer irritant effects and a greater preference compared with a tretinoin cream [62]. The preferred attributes reported for acne medications included: *easy to dispense/dispense the right amount*, *non-drying*, *product goes on/spreads smoothly*, *no residue*, and *creamy* [69].

Atopic dermatitis patients were shown to prefer creams [69]. When comparing different leave-on emollients, the patients valued *hydrating activity* (67%), and *greasiness* (51%), but not color or scent (*n* = 250) [74]. Atopic dermatitis patients considered the following condition-specific features relevant [69]: *is not noticeable to others/conceals area*, *good consistency*, *cooling*, *no residue, and soothing effect.* Attributes such as *easy to apply* (32%), *easy absorption* (6.8%), and *cooling effect* (6.8%) were also noted in a study involving both patients and caregivers (*n* = 103) [75]. Topical treatments were consistently described as being *greasy* and/or *messy*, inconvenient to carry or travel with, and time-consuming to apply. The burden described by both adolescents and caregivers in association with frequent topical-treatment administration was higher than for adults, highlighting the influence of demographics on patient preferences. Faster *dermal absorption* and the opportunity to test samples were mentioned by adolescents (*n* = 15) as preferences regarding treatment-specific attributes [76].

Systematic reviews summarize the findings of all the relevant individual studies and thus provide a higher level of evidence. A systematic review that addressed atopic dermatitis patients and caregivers found that the main preference factors for topical medicines were *odorless treatments*, *low visibility*, and *sparing use*, with little impact on daily life [77]. However, these preferences were supported by low-certainty evidence when compared with concerns about adverse effects. Fear of side effects, such as steroid phobia, can result in non-adherence to medication; this is a major issue to be addressed in the patient-centric design process by carefully selecting the drug and designing the vehicle to minimize the most troublesome adverse skin effects [78].

Patients with **rosacea** were reported to be neutral regarding their current treatments [79,80] but frequently reported concerns, such as efficacy and side effects, were not associated with treatment satisfaction (*n* = 216) [80]. Concerns about topical treatments rather than preferences regarding topical attributes were evaluated in these studies and, thus, specific preferences were not established. *Application residue* and, less frequently, *smell* or *texture* were rated as formulation-dependent concerns by a minority of the patients. More tolerable topical treatments that do not elicit burning, itch, and dryness were identified as unmet needs. Foams with azelaic acid have been studied as therapeutic alternatives to hydrogels, with azelaic acid or metronidazole showing good tolerability and efficacy [79,80].

Satisfaction with topical treatment and vehicle preference has been more extensively studied for **psoriasis**, probably because is a chronic disease with high prevalence and a low treatment-adherence rate [81]. Psoriasis patients (*n* = 17) have shown preferences for creams, ointments, and foams (particularly for the scalp) [69]. A small study on 20 patients showed that topical suspensions were preferred to ointments [82], which was consistent with other findings demonstrating a low level of satisfaction with treatment with *messy* ointments [83]. One of the attributes that were significantly highly rated for the suspension was comfort under clothing. In other studies, possible solutions suggested by patients with psoriasis to increase their satisfaction with topical treatments were *less greasy*, *sticky*, and *smelly formulations* [8,67]. The use of corticosteroid solutions by psoriasis patients has been proposed as a good alternative for patients who dislike greasy preparations, although these solutions are sometimes associated with burning or stinging. When spray-on solutions are overly expensive for patients, a possible alternative is to place a generic corticosteroid solution in an inexpensive spray bottle. When alcohol-based solutions cause excessive stinging, an oily vehicle can be prescribed for spray-on application [84]. Solution- and foam-based corticosteroid vehicles were also preferred to ointments, gels, and creams in a small study (*n* = 20) [83]. Adam et al. performed a retrospective study and analyzed the impact of changing drug bases for psoriasis from ointment or gel to aerosol foam, and they found a successful transition in 85% of the patients, with improved treatment adherence and better quality of life [85]. Foam bases were also preferred by plaque-psoriasis patients as easy-to-use topical-drug options [86,87,88,89]. Emerging vehicles/bases for psoriasis treatment are continuously investigated. New hydrophilic vehicles obtained with PAD technology protect drugs against hydrolysis, ensuring the stability of the calcipotriene/betamethasone combination while being more patient-friendly than current formulations for psoriasis treatment [90].

The three most highly valued attributes of topical products noted by psoriasis patients were as follows: *allow dressing shortly after application*, *good moisturizing properties*, and *use only once daily*. These were followed by *good absorption*, *does not leave stains, does not cause itching or burn*, and *does not run-off* [91]. These findings were consistent with those of another study, which highlighted *ease of application*, the *time needed for application*, the *cost of replacing stained clothes and bed linen*, *absorption*, and *messiness* as important characteristics for patient use [83]. A systematic review (*n* = 12) on psoriasis patients’ preferences regarding topical treatments found that overall, the patients preferred medicines that are *easy to apply*, *less messy*, and *have a pleasant scent* [67]. This review also emphasized that there is no single topical-drug product that suits everyone, as well as the importance of shared decision making.

The attributes that were reported simultaneously by patients with plaque psoriasis, atopic dermatitis, and acne were absorbed/disappears/dries quickly, available in various formulations, does not bleach or stain skin/hair/clothing, is not greasy/oily, is not sticky/tacky, is long-lasting/long-acting/stays on/lasts through sweating or hand washing, is fragrance- or odor-free, is easy to apply/simple to use, can use all the time, and moisturizing [69]. However, the ranking of these attributes in terms of importance was not reported.

For **seborrheic dermatitis**, little information is available, A ketoconazole-foam formulation for the treatment of seborrheic dermatitis was included in a more integrated analysis aiming to demonstrate that foams are preferable to other topical vehicles (*n* = 3398). The proportion of dermatological patients who preferred foam over other vehicles used in the past was greater than 60% when compared to cream, 70% compared to gel, and 60% compared to ointment [92].

The results obtained from the studies assessing vehicle preference are, however, limited by the numbers of vehicles compared, which are usually low, and the small sample sizes. Many studies on patient preferences regarding topical vehicles compared only two vehicles [62,82] or relied on patient perspectives/beliefs rather than experiences of using the vehicle [70]. The identification of dosage form also needs to be properly described. For instance, a gel can refer to a hydrogel or an oleogel, which have very different properties.

From the point of view of drug-product design, studying the topical attributes deemed most relevant by patients is meaningful and provides a rational basis for drug-product design. More studies, with larger sample sizes, addressing other skin conditions, and of good methodological quality are needed [93]. Systematic reviews for each skin condition would be highly useful. Based on a review of current studies on preferences regarding topical treatments Gutknecht et al. recommended that preferences have to be recorded in such a way that they are representative of the affected patients. Questions should be also asked comprehensibly and openly, and the options described should be realistic [93].

For pigmentary disorders, information on patient preferences is scarce. Combi-kits with sunscreen day cream and night cream were found to be very convenient, helping users to remember to apply the medication [94]. New drug-delivery systems for vitiligo treatment were proposed based on phospholipid-based carrier systems, which are thought to improve skin penetration and increase drug localization while putatively improving adherence because of their moisturizing effect, favorable rheological properties, and reduced side effects [95]. Lecithin organogels are among the phospholipid-based approaches studied for vitiligo treatment.

Preferences cannot be predicted by a single variable, such as demographics; hence, more clinical studies are needed to better understand the preferences of patients suffering from skin disorders [12]. From the industrial point of view, commercializing individualized products is not feasible. A product that meets every patient’s expectations is also practically impossible to achieve, since preferences often vary between individuals [96]. Patient interviews can be performed before defining the treatment regimen. Giving patients the option of participating in their choice of medication could prove critical to treatment adherence and, ultimately, clinical efficacy. From the point of view of healthcare practice, one possible way to meet patients’ preferences for topical vehicles is to allow them to try samples before establishing the treatment regimen. Pharmaceutical compounding also plays a key role in obtaining individualized medicines that are not available in the market [97]. The process of patient-centric compounding design was previously proposed [97], supported by close interactions between the patient, clinician, and pharmacist.

### 3.2. Target Product Profile (TPP)

The definition of the drug-product profile should take into account the needs and preferences of a given patient population and then translate this information into a profile that is as universal as possible. The drug product is considered the presentation of the topical treatment to the end user (patient/caregiver/health care provider) and includes the vehicle/base, formulation composition, dose, dosing frequency, primary, secondary, and tertiary packaging, dosing devices, and instructions for use. The triad of disease needs + patient needs + patient preferences is the cornerstone of the PCDPD process. Considerations regarding the packaging of topical products and TPP for selected skin disorders are addressed below.

#### 3.2.1. Packaging

The packages conventionally used for semisolid topical products are mainly tubes. Packages or applicators that ease the application by avoiding the use of the hands (sometimes called “no-mess applicators”) were recently introduced to the market. The avoidance of the use of hands during rub-in decreases the time spent on washing hands and the putative discomfort of the residue on the hands. Other devices that are used to help to define the amount to be applied in lesions are also available [98].

Recent technological advances, such as 3-D printing, offer an unlimited number of possibilities regarding package design. Instructions for use and for defining the correct amount to be used (e.g., the size of a pea, a finger-tip unit) can also be included within the package, which contributes to the education of patients and improves adherence [61].

#### 3.2.2. Target Product Profile for Selected Skin Disorders

Skin disorders were grouped according to the symptomatology and type of lesion, and a generic TPP was proposed for each illustrative disorder. All of the disorders had a general inflammatory character associated with some skin lesions and symptoms that differentiated them.

In general, the vehicles/bases used should be non-irritant and easy to spread to avoid friction. Many skin conditions, such as eczematous disorders, occur with inflammation, exhibiting erythema and edema.

**Figure 2:**
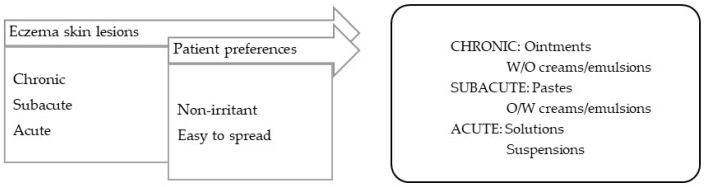
TPP for formulations for eczema-lesion treatment and most suitable vehicles/bases.

**Eczema** is characterized by inflammatory lesions of diverse etiology but with similar characteristics: erythema, vesicles, and desquamation. Different phases can be distinguished in eczema, and so the vehicles/bases should be selected accordingly: (a) in acute phase with erythema, vesicles, exudation, drying vehicles with absorbing capacity, such as suspensions, are preferred; (b) in subacute phase with peeling, excoriation, crusts, and, often, secondary infection, the vehicles should have non-occlusive characteristics, such as those of pastes (e.g., calamine lotion or zinc paste); (c) in the chronic phases of dry lesions, such as lichenification (thickening, darkening, skin folding), the vehicles should have a more occlusive and emollient capacity, such as that of ointments (Figure 2) [99].

1. Scaly and xerotic disorders.

**Figure 3:**
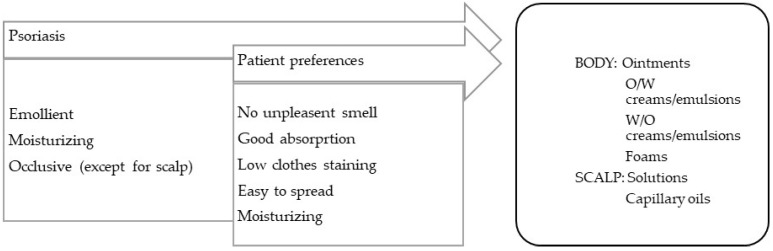
TPP for formulations for psoriasis treatment and most suitable vehicles/bases.

Formulations for disorders with scaliness and dry skin should be, in general, occlusive, lubricating, and emollient (Figure 3). The skin conditions in this category include psoriasis, ichthyosis, keratosis pillaris, and xerosis.

**Psoriasis** is a chronic erythematous–squamous disease with a high psychosocial impact. Lesions, or psoriatic plaques, present erythema, infiltration, and flaking. Plaque psoriasis, the most common form of the disease, can affect extensive areas of the skin, scalp, and nails. Itching and local pain are symptoms that are frequently reported by patients. Other forms of psoriasis are known, such as flexural psoriasis (on areas of sensitive skin), guttate psoriasis (after streptococcal infections), pustular psoriasis (featuring the presence of pustules, which are generally palmoplantar), erythrodermic psoriasis (a severe and generalized form of erythema), and arthropathic psoriasis (associated with inflammation of the joints, particularly the hands and feet) [99].

2. Long-term inflammatory disorders.

**Figure 4:**
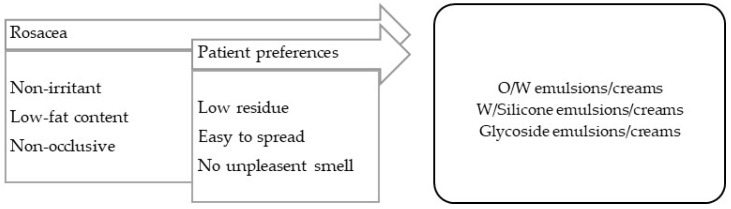
TPP for formulations for rosacea treatment and most suitable vehicles/bases.

**Rosacea** is essentially a form of facial inflammatory dermatitis characterized by erythema and, in a more advanced stage, by papulopustular lesions [99]. The vehicles/bases for rosacea treatment should have low fat contents and be non-irritant (Figure 4).

3. Seborrheic disorders.

In general, the vehicles/bases used should have low fat contents and not leave skin residue. Seborrheic conditions occur in high-sebaceous-gland-density locations, such as the face, trunk, or scalp, and include seborrheic dermatitis and acne.

**Figure 5:**
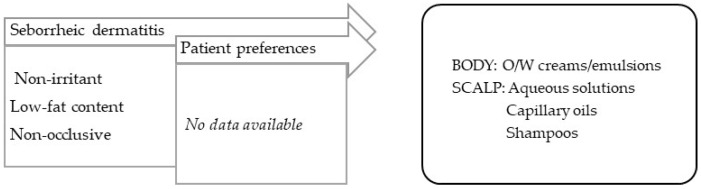
TPP for formulations for treatment of seborrheic dermatitis and most suitable vehicles/bases.

**Seborrheic dermatitis** is a form of chronic inflammatory dermatitis located in areas with an excess of sebum and a high prevalence of *Malassezia furfur* [99]. For applications on the body, O/W creams or emulsions low in fat should be used. For the scalp, O/W emulsions are less appealing, since they require clothing to be protected; instead surfactant-based shampoos should be used for washing and treatment, as well as capillary oils with silicones, which are slightly oily and confer emollience, and aqueous solutions for a drying effect (Figure 5).

**Figure 6:**
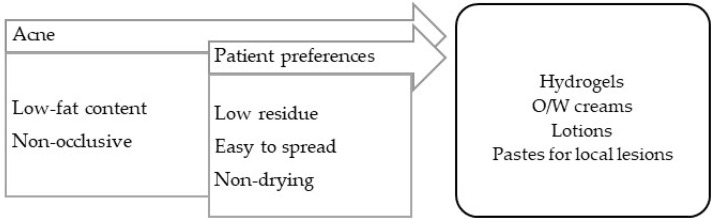
TPP for formulations for acne treatment and most suitable vehicles/bases.

**Acne** is an inflammation of the sebaceous glands with bacterial colonization (*Cutibacterium acnes*). Its lesions differ in severity and evolve, and include hyperkeratosis and comedones (non-inflammatory), papules and erythema (inflammatory), pustules and cysts (pustular), nodules, and scars (cicatricial) [99]. It should be noted that the vehicles for this pathology must contain reduced fat contents, and therapeutic practice is slightly complex because it often resorts to the use of drugs (Figure 6).

4. Pruritic disorders.

Pruritus is a common symptom that is widely spread in many diseases, not only of cutaneous origin, but also of systemic origin (neurologic, psychiatric, endocrine, hematologic, and others). Pruritus skin lesions present additional symptoms, which may include inflammation (erythema, edema), or dry and scaly skin—both of which may present with excoriations caused by scratching and lichenification, if chronic [99]. The vehicles and bases for these conditions should be adapted to the prevalent symptoms.

Skin conditions such as atopic dermatitis and urticaria are illustrative of pruritic dermatosis.

**Figure 7:**
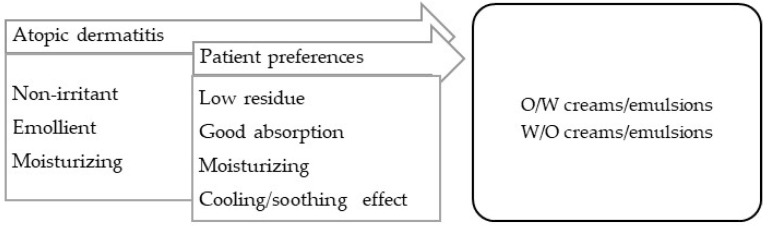
TPP for formulations for treatment of atopic dermatitis and most suitable vehicles/bases.

**Atopic dermatitis** is a chronic inflammatory dermatitis associated with intense pruritus [99]. In the acute phase, and if there is edema, the vehicle must be siccative (such as aqueous solutions), and when the exudation ceases, the vehicle can be changed to emulsions with a different fat contents, according to the occlusive effect required. Atopic dermatitis requires maintenance, in which the hydration of the skin is essential; numerous emulsified vehicles and oils can be enriched with moisturizing substances (Figure 7).

5. Pigmentation disorders.

**Figure 8:**
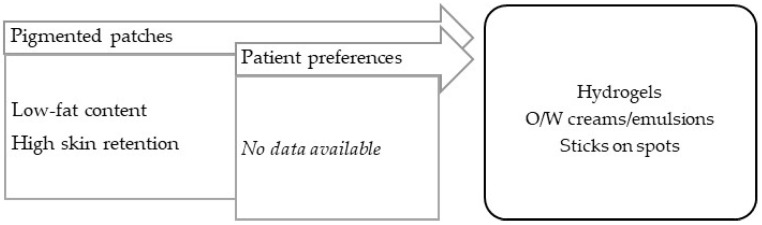
TPP for formulations for treatment of pigmentation disorders and most suitable vehicles/bases.

Hyperpigmentation (dark macules) results from an increase in melanin production or in the proliferation of melanocytes, originating in epidermal or dermal melanin deposition. The absence of local melanocytes leads to vitiligo, an hypopigmentary skin (white macules) disorder, possibly autoimmune in origin. In addition to a certain inflammatory grade, both lesions are characterized by apparently normal skin with no other symptoms, although they differ in terms of their extension [99]. A variety of vehicles/bases can be applied to ensure: (a) the vehiculation of several types of drug, (b) a non-irritation effect to counterbalance some sensitizing substances, and (c) adaptation to the location and extension of lesions. The most commonly used vehicles/bases are emulsions, creams, and stick bars for easy use (Figure 8).

The treatment of dermatoses with mixed symptomatology must target the most troublesome symptoms at the point of treatment prescription, in association with the patient’s specific skin condition and comorbidities. In all cases, the patient’s needs should be accounted for when prescribing the treatment regimen. These include visual, motor, and cognitive impairments, as well as poor hand sensitivity and the need for help in applying the treatment.

## 4. Conclusions

Topical medicines have been associated with poor adherence despite being considered the mainstay of dermatological treatments.

Patient-centric drug-product pharmaceutical design can be a useful tool to improve adherence in dermatology by taking into consideration both the disease and the patient’s needs and preferences to improve the acceptability of the drug product. The target profile of the drug product, based on the lesions’ characteristics and location, the symptomatology of the underlying skin disease, and the patient’s preferences, supports the selection of the most appropriate dosage form and formulation composition. The systematization of target product profiles provided herein can help members of the pharmaceutical industry to offer topical drug products with more universal profiles. Furthermore, it also of utmost importance to clinicians to support the selection of the most suitable topical medicine, as well as the prescription of customized compounding formulations. After optimized formulations are obtained, they should be characterized in terms of the features that are relevant from the patient’s perspective to confirm their suitability with the TPP, or the need for reformulation. Increases in patient satisfaction with topical treatments should also be verified in comparison with standard treatments in a sample population. Patient-centric design, however, should not be regarded as a single intervention, but rather as a strategy that complements other interventions aimed at improving medication adherence.

The main purpose of vehicles is to ensure drugs’ stability and their delivery, in therapeutic doses, to the sites of action. After establishing the most suitable vehicle/base, the final decision should be centered on the patient’s preferences, since it is certain that a drug product will not be effective if it is not used. Medicine rejection may be countered by tailoring vehicles to individualized patient preferences. Many formulations are available to help clinicians to prescribe customized treatments. Clinicians can also rely on the technical expertise of pharmacists. Allowing patients to try samples of different vehicles before establishing the treatment regimen can be also very helpful, especially when the identification of patient preferences is troublesome.

Insights regarding the development of new vehicles with better organoleptic features, as well as new studies on patient preferences and on the therapeutic effects of topical vehicles on clinical outcomes, need to be continuously analyzed and translated in the update of the TPPs for the most common skin disorders.

## Figures and Tables

**Table 1:** Characteristics of liquid vehicles [15,16].

Vehicle	Composition	Characteristics	Examples
Solutions	Solute (or solutes) dissolved in a liquid solvent such as water, alcohol, glycols, or oil	Clear and transparent	Urea aqueous solution
Suspensions	Insoluble powders dispersed in a liquid phase	Translucent or opaque; with time, the suspended solids tend to settle	Aqueous zinc suspension
Emulsions	Homogeneous two-phase liquid systems of water, liquid oils, and emulsifying agents	Opaque and homogeneous	Lanette lotion

**Table 2:** Characteristics of semisolid bases [15,16].

Base	Composition	Characteristics	Examples
Hydrophobic	Solid and liquid paraffin, petrolatum, waxes, triglycerides, vegetable oils, silicone oils	Emollients, occlusive, greasy, and difficult to remove; high retention on the skin; form an occlusive layer that prevents water loss; rapid delivery of hydrophilic drugs; very low water absorption (<5%)	Coal-tar-paraffin ointment
Absorption	Water-absorbent components: lanolin; lanolin alcohols; cholesterol; bees wax; emulsifying agents	Emollient, occlusive, and greasy, albeit less than hydrophobic bases; make emulsions by adding water;	Hydrophilic ointment (USP) or Cetylic ointment (PPh)
Water-in-oil (W/O) cream	Water, hydrocarbons, waxes, polyethylene glycols, and emulsifying agents	Water-in-oil two-phase system; occlusive properties; when applied to the skin, they leave an oily film on the surface of the skin	Cold cream (USP) Cooling ointment (FNA)
Oil-in-water (O/W) cream	Water, hydrocarbons, waxes, polyethylene glycols, and emulsifying agents	Oil-in-water two-phase system; non-occlusive; non-greasy; moisturizing and emollient effect	Lanette cream (BP)
Anhydrous hydrophilic	PEGs of different molecular weights	Non-occlusive, non-oily, and easily removable; exudate miscibility but hyperosmotic miscible; low water absorption	Macrogol ointment
Hydrogels	Organic macromolecules or polymers dispersed in water: natural (xanthan gum, alginate); semi-synthetics (cellulose derivatives); synthetics (carbomer)	Transparent aqueous formulations with no grease; easy to apply and remove; refreshing properties; little moisturizing or emollient effect; may contain alcohol, so they are likely to cause irritation and have drying ability; the aqueous medium is susceptible to degradation	Carbomer gel Carboxymethylcellulose gel
Oleogels	Organic macromolecules or polymers dispersed in a lipophilic oil	Moisturizing or emollient effect; leave an oily film on the surface of the skin	12-hydroxystearic acid Oleogel
Pastes	Insoluble drug in an ointment or a hydrophilic base	High content of insoluble powders; protective effect; drying effect; varied consistency and solubility	Zinc-oxide paste Darier paste
Foams	Nonpolar hydrocarbons as propellants; Solvents include water, oils, ethanol, acetone, hexadecyl alcohol, glycol ethers, and polyglycols	Pressurized solutions or fluid emulsions mixed with a propellent; no need to spread the product and quick-drying; low skin residue low hydration or occlusive effect	Coal-tar foam (Scytera^®^)

USP, United States Pharmacopoeia; PPh, Portuguese Pharmacopoeia; PEG, Polyoxyethylene glycol; FNA, Nederland Formulary (Formularium der Nederlandse Apothekers); BP, British Pharmacopoeia.

**Table 3:** Vehicle/base selection according to the type of lesion and body area.

	Palmoplantar	Glabrous	Hairy	Facial or Intertrigital
**Very dry**	Hydrophobic ointments	Hydrophobic ointments	Oil solutions	W/O emulsions
	Absorption ointments	Absorption ointments	W/O emulsions	O/W emulsions
		W/O emulsions		W/S emulsions
**Dry**	Hydrophobic ointments	Absorption ointments	Oil solutions	W/O emulsions
	Absorption ointments	W/O emulsions	O/W emulsions	O/W emulsions
	W/O emulsions	O/W emulsions		W/S emulsions
**Subacute**	Hydrophobic ointments	W/O emulsions	Oil solutions	W/O emulsions
	W/O emulsions	O/W emulsions	Shampoos	O/W emulsions
	O/W emulsions	Hydrophobic pastes		W/S emulsions
**Wet**	W/O emulsions	O/W emulsions	O/W emulsions	O/W emulsions
	O/W emulsions		Hydrogels	Hydrogels
			Aqueous solutions	Hydrophilic pastes
**Exudative**	Hydrogels	Hydrogels	Hydrogels	Hydrogels
	Aqueous solutions	Aqueous solutions	Aqueous solutions	Aqueous solutions
	Hydrophilic pastes	Hydrophilic pastes		Hydrophilic pastes

W/O, water-in-oil; O/W, oil-in-water; W/S, water-in-silicone.

**Table 4:** Vehicle selection according to the facial-skin type.

Dry Skin	Oily Skin	Combination Skin	Sensitive Skin
W/O cream	O/W cream (<30% F.C.)	O/W cream (<30% F.C.)	O/W cream (>50% F.C.)
O/W cream (>50% F.C.)	W/S cream	W/S cream	Oils
	Emulgel Hydrogel	Glycoside cream	Glycoside cream Emulgel

W/O, water-in-oil; O/W, oil-in-water; W/S, water-in-silicone; F.C., fat content.

## Data Availability

No new data were created or analyzed in this study. Data sharing is not applicable to this article.

## References

[1] Ana Teixeira M.T., Almeida V., Almeida I.F., Costa E., Giardini A., Monaco A. (2017). Adherence to Topical Treatment in Psoriasis. Adherence to Medical Plans for Active and Healthy Ageing.

[2] Augustin M., Holland B., Dartsch D., Langenbruch A., Radtke M.A. (2011). Adherence in the treatment of psoriasis: A systematic review. Dermatology.

[3] Miyachi Y., Hayashi N., Furukawa F., Akamatsu H., Matsunaga K., Watanabe S., Kawashima M. (2011). Acne management in Japan: Study of patient adherence. Dermatology.

[4] Snyder A., Farhangian M., Feldman S.R. (2015). A review of patient adherence to topical therapies for treatment of atopic dermatitis. Cutis.

[5] Teixeira A., Oliveira C., Teixeira M., Rita Gaio A., Lobo J.M.S., de Almeida I.F.M., Almeida V. (2017). Development and Validation of a Novel Questionnaire for Adherence with Topical Treatments in Psoriasis (QATOP). Am. J. Clin. Dermatol..

[6] Tveit K.S., Duvetorp A., Østergaard M., Skov L., Danielsen K., Iversen L., Seifert O. (2019). Treatment use and satisfaction among patients with psoriasis and psoriatic arthritis: Results from the NORdic PAtient survey of Psoriasis and Psoriatic arthritis (NORPAPP). J. Eur. Acad. Dermatol. Venereol..

[7] Schaarschmidt M.L., Umar N., Schmieder A., Terris D.D., Goebeler M., Goerdt S., Peitsch W.K. (2013). Patient preferences for psoriasis treatments: Impact of treatment experience. J. Eur. Acad. Dermatol. Venereol..

[8] Fouéré S., Adjadj L., Pawin H. (2005). How patients experience psoriasis: Results from a European survey. J. Eur. Acad. Dermatol. Venereol..

[9] Brown K.K., Rehmus W.E., Kimball A.B. (2006). Determining the relative importance of patient motivations for nonadherence to topical corticosteroid therapy in psoriasis. J. Am. Acad. Dermatol..

[10] Teixeira A., Teixeira M., Almeida V., Gaio R., Torres T., Magina S., Cunha C., Sousa Lobo J.M., Almeida I.F. (2021). Does the Vehicle Matter? Real-World Evidence on Adherence to Topical Treatment in Psoriasis. Pharmaceutics.

[11] Puig L., Carrascosa J.M., Belinchón I., Fernández-Redondo V., Carretero G., Ruiz-Carrascosa J.C., Careaga J.M., de la Cueva P., Gárate M.T., Ribera M. (2013). Adherence and Patient Satisfaction With Topical Treatment in Psoriasis, and the Use, and Organoleptic Properties of Such Treatments: A Delphi Study With an Expert Panel and Members of the Psoriasis Group of the Spanish Academy of Dermatology and Venereology. Actas Dermo-Sifiliográficas.

[12] Iversen L., Jakobsen H.B. (2016). Patient Preferences for Topical Psoriasis Treatments are Diverse and Difficult to Predict. Dermatol. Ther..

[13] Council of Europe (2019). European Pharmacopoeia, 10th Edition 2020.

[14] Teixeira A., Vasconcelos V., Teixeira M., Almeida V., Azevedo R., Torres T., Sousa Lobo J.M., Costa P.C., Almeida I.F. (2019). Mechanical Properties of Topical Anti-Psoriatic Medicines: Implications for Patient Satisfaction with Treatment. AAPS PharmSciTech.

[15] Swarbrick J. (2007). Encyclopedia of Pharmaceutical Technology.

[16] Taylor K.M.G., Aulton M.E., Taylor K.M.G., Aulton M.E. (2021). Aulton’s Pharmaceutics: The Design and Manufacture of Medicines.

[17] Lukic M., Pantelic I., Savic S. (2016). An Overview of Novel Surfactants for Formulation of Cosmetics with Certain Emphasis on Acidic Active Substances. Tenside Surfactants Deterg..

[18] Pal A., Mondal M.H., Adhikari A., Bhattarai A., Saha B. (2021). Scientific information about sugar-based emulsifiers: A comprehensive review. RSC Adv..

[19] Ajazuddin, Alexander A., Khichariya A., Gupta S., Patel R.J., Giri T.K., Tripathi D.K. (2013). Recent expansions in an emergent novel drug delivery technology: Emulgel. J. Control. Release.

[20] Somasundaran P., Mehta S.C., Purohit P. (2006). Silicone emulsions. Adv. Colloid Interface Sci..

[21] Mancuso A., Tarsitano M., Udongo B.P., Cristiano M.C., Torella D., Paolino D., Fresta M. (2022). A comparison between silicone-free and silicone-based emulsions: Technological features and in vivo evaluation. Int. J. Cosmet. Sci..

[22] Ohsedo Y. (2022). N-Alkylhydantoins as New Organogelators and Their Ability to Create Thixotropic Mixed Molecular Organogels. Gels.

[23] Ambreen Z., Faran S.A., Daniel A., Khalid S.H., Khan I.U., Asif M., Rehman A., Mehmood H.Q., Asghar S. (2022). Physicochemical, rheological and antifungal evaluation of miconazole nitrate organogels for topical delivery. Pak. J. Pharm. Sci..

[24] Jun Yang S., Yoon K.S. (2021). Preparation and Evaluation of Pluronic Lecithin Organogels in Cosmetics. J. Cosmet. Sci..

[25] Kircik L.H. (2019). Vehicles Always Matter. J. Drugs Dermatol..

[26] Hoc D., Haznar-Garbacz D. (2021). Foams as unique drug delivery systems. Eur. J. Pharm. Biopharm..

[27] Daniels R., Knie U. (2007). Galenics of dermal products--vehicles, properties and drug release. J. Dtsch. Dermatol. Ges..

[28] Weiss S.C. (2011). Conventional topical delivery systems. Dermatol. Ther..

[29] Rosen J., Landriscina A., Friedman A.J. (2014). Principles and approaches for optimizing therapy with unique topical vehicles. J. Drugs Dermatol..

[30] Mayba J.N., Gooderham M.J. (2018). A Guide to Topical Vehicle Formulations. J. Cutan. Med. Surg..

[31] Barnes T.M., Mijaljica D., Townley J.P., Spada F., Harrison I.P. (2021). Vehicles for Drug Delivery and Cosmetic Moisturizers: Review and Comparison. Pharmaceutics.

[32] EMA Reflection Paper on the Use of Extrapolation in the Development of Medicines for Paediatrics (EMA/189724/2018). https://www.ema.europa.eu/en/documents/scientific-guideline/adopted-reflection-paper-use-extrapolation-development-medicines-paediatrics-revision-1_en.pdf.

[33] EMA Guideline on Pharmaceutical Development of Medicines for Paediatric Use (EMA/CHMP/QWP/805880/2012 Rev.2). https://www.ema.europa.eu/en/documents/scientific-guideline/guideline-pharmaceutical-development-medicines-paediatric-use_en.pdf.

[34] EMA Reflection paper on the pharmaceutical development of medicines for use in the older population (EMA/CHMP/QWP/292439/2017). https://www.ema.europa.eu/en/documents/scientific-guideline/reflection-paper-pharmaceutical-development-medicines-use-older-population-first-version_en.pdf.

[35] FDA Patient-Focused Drug Development: Methods to Identify What Is Important to Patients. https://www.fda.gov/media/131230/download.

[36] Perfetto E.M., Burke L., Oehrlein E.M., Epstein R.S. (2015). Patient-Focused Drug Development: A New Direction for Collaboration. Med. Care.

[37] Chalasani M., Vaidya P., Mullin T. (2018). Enhancing the incorporation of the patient’s voice in drug development and evaluation. Res. Involv. Engagem..

[38] Zvonareva O., Craveț C., Richards D.P. (2022). Practices of patient engagement in drug development: A systematic scoping review. Res. Involv. Engagem..

[39] ICH Proposed ICH guideline work to advance patient focused drug development. https://www.ema.europa.eu/en/documents/scientific-guideline/ich-reflection-paper-proposed-ich-guideline-work-advance-patient-focused-drug-development_en.pdf.

[40] Stegemann S., Ternik R.L., Onder G., Khan M.A., van Riet-Nales D.A. (2016). Defining Patient Centric Pharmaceutical Drug Product Design. AAPS J..

[41] Timpe C., Stegemann S., Barrett A., Mujumdar S. (2020). Challenges and opportunities to include patient-centric product design in industrial medicines development to improve therapeutic goals. Br. J. Clin. Pharmacol..

[42] Algorri M., Cauchon N.S., Christian T., O’Connell C., Vaidya P. (2023). Patient-Centric Product Development: A Summary of Select Regulatory CMC and Device Considerations. J. Pharm. Sci..

[43] Ogbonna J.D.N., Cunha E., Attama A.A., Ofokansi K.C., Ferreira H., Pinto S., Gomes J., Marx Í.M.G., Peres A.M., Lobo J.M.S. (2022). Overcoming Challenges in Pediatric Formulation with a Patient-Centric Design Approach: A Proof-of-Concept Study on the Design of an Oral Solution of a Bitter Drug. Pharmaceuticals.

[44] Shariff Z., Kirby D., Missaghi S., Rajabi-Siahboomi A., Maidment I. (2020). Patient-Centric Medicine Design: Key Characteristics of Oral Solid Dosage Forms that Improve Adherence and Acceptance in Older People. Pharmaceutics.

[45] Drumond N. (2020). Future Perspectives for Patient-Centric Pharmaceutical Drug Product Design with Regard to Solid Oral Dosage Forms. J. Pharm. Innov..

[46] Oliveira R.S., da Silva D.F., Mota S., Garrido J., Garrido E.M., Lobo J.M.S., Almeida I.F. (2022). Design of an Emulgel for Psoriasis Focused on Patient Preferences. Appl. Sci..

[47] Cook N.S., Cave J., Holtorf A.P. (2019). Patient Preference Studies During Early Drug Development: Aligning Stakeholders to Ensure Development Plans Meet Patient Needs. Front. Med..

[48] Stegemann S., Sheehan L., Rossi A., Barrett A., Paudel A., Crean A., Ruiz F., Bresciani M., Liu F., Shariff Z. (2022). Rational and practical considerations to guide a target product profile for patient-centric drug product development with measurable patient outcomes—A proposed roadmap. Eur. J. Pharm. Biopharm..

[49] Surber C., Smith E.W. (2005). The mystical effects of dermatological vehicles. Dermatology.

[50] Danby S.G., Draelos Z.D., Gold L.F.S., Cha A., Vlahos B., Aikman L., Sanders P., Wu-Linhares D., Cork M.J. (2022). Vehicles for atopic dermatitis therapies: More than just a placebo. J. Dermatol. Treat..

[51] Crowther J.M., Sieg A., Blenkiron P., Marcott C., Matts P.J., Kaczvinsky J.R., Rawlings A.V. (2008). Measuring the effects of topical moisturizers on changes in stratum corneum thickness, water gradients and hydration in vivo. Br. J. Dermatol..

[52] Spada F., Barnes T.M., Greive K.A. (2018). Skin hydration is significantly increased by a cream formulated to mimic the skin’s own natural moisturizing systems. Clin. Cosmet. Investig. Dermatol..

[53] Danby S.G., Andrew P.V., Taylor R.N., Kay L.J., Chittock J., Pinnock A., Ulhaq I., Fasth A., Carlander K., Holm T. (2022). Different types of emollient cream exhibit diverse physiological effects on the skin barrier in adults with atopic dermatitis. Clin Exp. Dermatol..

[54] Lodén M. (2012). Effect of moisturizers on epidermal barrier function. Clin. Dermatol..

[55] Draelos Z.D. (2012). New treatments for restoring impaired epidermal barrier permeability: Skin barrier repair creams. Clin. Dermatol..

[56] Murasawa Y., Furuta K., Noda Y., Nakamura H., Fujii S., Isogai Z. (2018). Ointment vehicles regulate the wound-healing process by modifying the hyaluronan-rich matrix. Wound Repair Regen..

[57] van Zuuren E.J., Fedorowicz Z., Christensen R., Lavrijsen A., Arents B.W.M. (2017). Emollients and moisturisers for eczema. Cochrane Database Syst. Rev..

[58] Spigariolo C.B., Ferrucci S.M. (2023). Efficacy and tolerability of a repairing moisturizing cream containing amino-inositole and urea 10% in adults with chronic eczematous dermatitis of the hands. Ital. J. Dermatol. Venerol..

[59] Fakhouri T., Yentzer B.A., Feldman S.R. (2009). Advancement in benzoyl peroxide-based acne treatment: Methods to increase both efficacy and tolerability. J. Drugs Dermatol..

[60] Hoffman L.K., Bhatia N., Zeichner J., Kircik L.H. (2018). Topical Vehicle Formulations in the Treatment of Acne. J. Drugs Dermatol..

[61] Menditto E., Orlando V., De Rosa G., Minghetti P., Musazzi U.M., Cahir C., Kurczewska-Michalak M., Kardas P., Costa E., Sousa Lobo J.M. (2020). Patient Centric Pharmaceutical Drug Product Design-The Impact on Medication Adherence. Pharmaceutics.

[62] Draelos Z., Tanghetti E., Guenin E. (2022). Vehicle Formulation Impacts Tolerability and Patient Preference: Comparison of Tretinoin Branded Lotion and Generic Cream. J. Drugs Dermatol..

[63] Tan X., Feldman S.R., Chang J., Balkrishnan R. (2012). Topical drug delivery systems in dermatology: A review of patient adherence issues. Expert Opin. Drug Deliv..

[64] Patel N.U., D’Ambra V., Feldman S.R. (2017). Increasing Adherence with Topical Agents for Atopic Dermatitis. Am. J. Clin. Dermatol..

[65] Umar N., Yamamoto S., Loerbroks A., Terris D. (2012). Elicitation and use of patients’ preferences in the treatment of psoriasis: A systematic review. Acta Derm. Venereol..

[66] de Wijs L.E.M., van Egmond S., Devillers A.C.A., Nijsten T., Hijnen D., Lugtenberg M. (2023). Needs and preferences of patients regarding atopic dermatitis care in the era of new therapeutic options: A qualitative study. Arch. Dermatol. Res..

[67] Svendsen M.T., Feldman S.R., Tiedemann S.N., Sørensen A.S.S., Rivas C.M.R., Andersen K.E. (2021). Psoriasis patient preferences for topical drugs: A systematic review. J. Dermatol. Treat..

[68] Park E.-K., Song K.-W. (2010). Rheological evaluation of petroleum jelly as a base material in ointment and cream formulations: Steady shear flow behavior. Arch. Pharmacal Res..

[69] Eastman W.J., Malahias S., Delconte J., DiBenedetti D. (2014). Assessing attributes of topical vehicles for the treatment of acne, atopic dermatitis, and plaque psoriasis. Cutis.

[70] Figenshau K., Kimmis B.D., Reicherter P. (2020). Variations in preference for topical vehicles among demographic groups. Cutis.

[71] Fisher E.J., Adams B.B. (2008). African American and Caucasian patients’ vehicle preference for the scalp. J. Am. Acad. Dermatol..

[72] Kircik L.H., Green L., Guenin E., Khalid W., Alexander B. (2022). Dermal sensitization, safety, tolerability, and patient preference of tazarotene 0.045% lotion from five clinical trials. J. Dermatolog. Treat..

[73] Kellett N., West F., Finlay A.Y. (2006). Conjoint analysis: A novel, rigorous tool for determining patient preferences for topical antibiotic treatment for acne. A randomised controlled trial. Br. J. Dermatol..

[74] Kunkiel K., Natkańska A., Nędzi M., Zawadzka-Krajewska A., Feleszko W. (2022). Patients’ preferences of leave-on emollients: A survey on patients with atopic dermatitis. J. Dermatol. Treat..

[75] Ervin C., Crawford R., Evans E., Feldman S.R., Zeichner J., Zielinski M.A., Cappelleri J.C., DiBonaventura M., Takiya L., Myers D.E. (2022). Patient and caregiver preferences on treatment attributes for atopic dermatitis. J. Dermatol. Treat..

[76] Kosse R.C., Bouvy M.L., Daanen M., de Vries T.W., Koster E.S. (2018). Adolescents’ Perspectives on Atopic Dermatitis Treatment-Experiences, Preferences, and Beliefs. JAMA Dermatol..

[77] Maleki-Yazdi K.A., Heen A.F., Zhao I.X., Guyatt G.H., Suzumura E.A., Makhdami N., Chen L., Winders T., Wheeler K.E., Wang J. (2023). Values and Preferences of Patients and Caregivers Regarding Treatment of Atopic Dermatitis (Eczema): A Systematic Review. JAMA Dermatol..

[78] Contento M., Cline A., Russo M. (2021). Steroid Phobia: A Review of Prevalence, Risk Factors, and Interventions. Am. J. Clin. Dermatol..

[79] Williamson T., Cameron J., McLeod K., Turner B., Quillen A., LaRose A. (2018). Patient Concerns and Treatment Satisfaction in Patients Treated with Azelaic Acid Foam for Rosacea. SKIN J. Cutan. Med..

[80] Williamson T., Cheng W.Y., McCormick N., Vekeman F. (2018). Patient Preferences and Therapeutic Satisfaction with Topical Agents for Rosacea: A Survey-Based Study. Am. Health Drug Benefits.

[81] Damiani G., Bragazzi N.L., Karimkhani Aksut C., Wu D., Alicandro G., McGonagle D., Guo C., Dellavalle R., Grada A., Wong P. (2021). The Global, Regional, and National Burden of Psoriasis: Results and Insights From the Global Burden of Disease 2019 Study. Front. Med..

[82] Sandoval L., Huang K., Harrison J., Clark A., Feldman S. (2014). Calcipotriene 0.005%-Betamethasone Dipropionate 0.064% Ointment Versus Topical Suspension in the Treatment of Plaque Psoriasis: A Randomized Pilot Study of Patient Preference. Cutis.

[83] Housman T.S., Mellen B.G., Rapp S.R., Fleischer A.B., Feldman S.R. (2002). Patients with psoriasis prefer solution and foam vehicles: A quantitative assessment of vehicle preference. Cutis.

[84] Hill D., Farhangian M.E., Feldman S.R. (2016). Increasing adherence to topical therapy in psoriasis through use of solution medication. Dermatol. Online J..

[85] Adam D.N., Abdulla S.J., Fleming P., Gooderham M.J., Ashkenas J., McCracken C.B. (2022). Transition of Topical Therapy Formulation in Psoriasis: Insights from a Canadian Practice Reflective. Skin Therapy Lett..

[86] Chung M., Yeroushalmi S., Hakimi M., Bartholomew E., Liao W., Bhutani T. (2022). A critical review of halobetasol propionate foam (0.05%) as a treatment option for adolescent plaque psoriasis. Expert Rev. Clin. Immunol..

[87] Bhatia N., Stein Gold L., Kircik L.H., Schreiber R. (2019). Two Multicenter, Randomized, Double-Blind, Parallel Group Comparison Studies of a Novel Foam Formulation of Halobetasol Propionate, 0.05% vs Its Vehicle in Adult Subjects With Plaque Psoriasis. J Drugs Dermatol..

[88] Aschoff R., Bewley A., Dattola A., De Simone C., Lahfa M., Llamas-Velasco M., Martorell A., Pavlovic M., Sticherling M. (2021). Beyond-Mild Psoriasis: A Consensus Statement on Calcipotriol and Betamethasone Dipropionate Foam for the Topical Treatment of Adult Patients. Dermatol. Ther..

[89] Dattola A., Silvestri M., Bennardo L., Passante M., Rizzuto F., Dastoli S., Patruno C., Bianchi L., Nisticò S.P. (2020). A novel vehicle for the treatment of psoriasis. Dermatol. Ther..

[90] Pinter A., Green L.J., Selmer J., Praestegaard M., Gold L.S., Augustin M. (2022). A pooled analysis of randomized, controlled, phase 3 trials investigating the efficacy and safety of a novel, fixed dose calcipotriene and betamethasone dipropionate cream for the topical treatment of plaque psoriasis. J. Eur. Acad. Dermatol. Venereol..

[91] Vasconcelos V., Teixeira A., Almeida V., Teixeira M., Ramos S., Torres T., Sousa Lobo J.M., Almeida I.F. (2019). Patient preferences for attributes of topical anti-psoriatic medicines. J. Dermatol. Treat..

[92] Weiss S., Wyres M., Brundage T. (2011). A novel foam vehicle is consistently preferred by patients for dermatologic conditions. J. Am. Acad. Dermatol..

[93] Gutknecht M., Schaarschmidt M.L., Herrlein O., Augustin M. (2016). A systematic review on methods used to evaluate patient preferences in psoriasis treatments. J. Eur. Acad. Dermatol. Venereol..

[94] Nair P.A., Vora R.V., Jivani N.B., Gandhi S.S. (2021). A study of clinical profile and quality of life in patients with scabies. Int. J. Res. Dermatol..

[95] Garg B.J., Saraswat A., Bhatia A., Katare O.P. (2010). Topical treatment in vitiligo and the potential uses of new drug delivery systems. Indian J. Dermatol. Venereol. Leprol..

[96] Felix K., Unrue E., Inyang M., Cardwell L.A., Oussedik E., Richardson I., Feldman S.R. (2020). Patients preferences for different corticosteroid vehicles are highly variable. J. Dermatol. Treat..

[97] Carvalho M., Almeida I.F. (2022). The Role of Pharmaceutical Compounding in Promoting Medication Adherence. Pharmaceuticals.

[98] Savary J., Ortonne J.P., Aractingi S. (2005). The right dose in the right place: An overview of current prescription, instruction and application modalities for topical psoriasis treatments. J. Eur. Acad. Dermatol. Venereol..

[99] Buxton P.K., Morris-Jones R., Paul K., Buxton R.M.-J. (2013). ABC of Dermatology.

